# Widespread bilateral lung, liver, and spleen hydatid cysts

**DOI:** 10.1590/0037-8682-0211-2022

**Published:** 2022-08-12

**Authors:** Yener Aydin, Nurhak Aksungur, Ali Bilal Ulas

**Affiliations:** 1 Ataturk University, Medical Faculty, Department of Thoracic Surgery, Erzurum, Turkey.; 2 Ataturk University, Medical Faculty, Department of General Surgery, Erzurum, Turkey.

**FIGURE 1:**
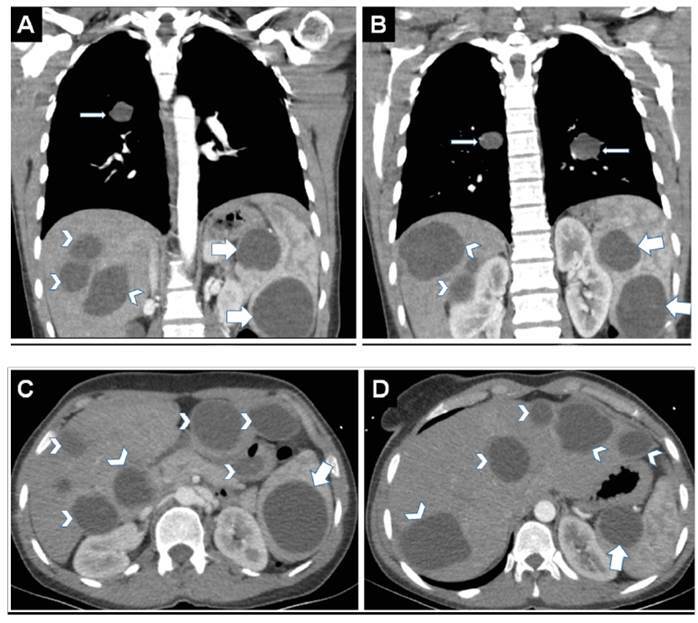
**(AB)** Approximately 4 cm in diameter in the left lower, right lower, and upper lobes in the lung (thin arrows); **(A-D)** in the liver, the largest cyst is approximately 6 cm in diameter in the eighth segment, multiple hypodense in both lobes, well-defined and non-enhancing (arrowheads); **(A-D)** two cystic lesions, the largest of which is 6.5 cm in diameter, are observed in the spleen (thick arrow).

A 31-year-old female patient presented with complaints of abdominal pain, cough, and hemoptysis. Multiple cystic lesions were detected in the right lower, right upper, and left lower lobes of the lungs; right and left lobes of the liver; and spleen (Figure 1). The patient underwent surgery for bilateral lung, liver, and spleen hydatid cysts.

Hydatid cyst is a parasitic disease caused by *Echinococcus granulosus* that has been well known since ancient times^1^. The most common organs affected by this disease are the liver, lungs, and spleen. The diagnosis of hydatid cyst is usually made by serological tests such as immunoglobulin G enzyme-linked immunoassay, hemagglutination, and imaging techniques including ultrasonography, computed tomography, and magnetic resonance imaging^2^. The majority of pulmonary hydatid cysts require surgical treatment. Treatment of hydatid cysts involving both the liver and spleen can be performed surgically in the same session^3^. In patients with hydatid cysts with multiorgan involvement, anthelmintics such as albendazole or mebendazole should be administered to reduce postoperative recurrences.
